# Personalised Medicine for Tuberculosis and Non-Tuberculous Mycobacterial Pulmonary Disease

**DOI:** 10.3390/microorganisms9112220

**Published:** 2021-10-26

**Authors:** Kartik Kumar, Onn Min Kon

**Affiliations:** 1 National Heart and Lung Institute, Imperial College London, Dovehouse Street, London SW3 6LY, UK; kartik.kumar@nhs.net; 2 Department of Respiratory Medicine, St Mary’s Hospital, Imperial College Healthcare NHS Trust, Praed Street, London W2 1NY, UK

**Keywords:** personalised medicine, tuberculosis, non-tuberculous mycobacteria, risk factors, nucleic acid amplification assays, next generation sequencing, host-directed therapies, therapeutic drug monitoring, biomarkers

## Abstract

Personalised medicine, in which clinical management is individualised to the genotypic and phenotypic data of patients, offers a promising means by which to enhance outcomes in the management of mycobacterial pulmonary infections. In this review, we provide an overview of how personalised medicine approaches may be utilised to identify patients at risk of developing tuberculosis (TB) or non-tuberculous mycobacterial pulmonary disease (NTM-PD), diagnose these conditions and guide effective treatment strategies. Despite recent technological and therapeutic advances, TB and NTM-PD remain challenging conditions to diagnose and treat. Studies have identified a range of genetic and immune factors that predispose patients to pulmonary mycobacterial infections. Molecular tests such as nucleic acid amplification assays and next generation sequencing provide a rapid means by which to identify mycobacterial isolates and their antibiotic resistance profiles, thus guiding selection of appropriate antimicrobials. Host-directed therapies and therapeutic drug monitoring offer ways of tailoring management to the clinical needs of patients at an individualised level. Biomarkers may hold promise in differentiating between latent and active TB, as well as in predicting mycobacterial disease progression and response to treatment.

## 1. Background

Mycobacterial lung diseases impose a significant healthcare and socioeconomic burden globally. In 2019, worldwide there were 7.1 million new diagnoses of tuberculosis (TB); 206,030 notified cases of rifampicin-resistant/multidrug-resistant (MDR) TB; 208,000 TB deaths among people living with human immunodeficiency virus (HIV) infection and 1.2 million deaths attributable to TB among HIV-negative people [1]. Non-tuberculous mycobacteria (NTM), which refer to all mycobacterial species other than *Mycobacterium tuberculosis* and *M. leprae*, may cause significant lung disease known as NTM pulmonary disease (NTM-PD). NTM-PD incidence rose from 3.13/100,000 to 4.73/100,000 between 2008 and 2015 in the USA and NTM infection incidence increased from 1.0/100,000 to 17.9/100,000 between 2003 and 2016 in the Republic of Korea, with the rises highest among females and older age groups [2,3]. While the human population is the reservoir of *M. tuberculosis* in endemic countries and regions, NTM are ubiquitous environmental organisms. The incidence of NTM species varies in different regions, with a higher diversity found in humid and temperate climates [4]. Many challenges persist in the management of TB and NTM-PD [5,6]. Diagnosis is complex, requiring a combination of clinical, microbiological and radiological considerations [7,8]. Clinical symptoms may be non-specific, comprising any combination of breathlessness, cough, sputum production, haemoptysis, fever, night sweats, weight loss and fatigue. While culture-based techniques remain the mainstay of identifying mycobacterial isolates, they are associated with limitations, including the slow rate of growth of organisms and the potential for environmental contamination. TB and NTM-PD are also difficult to treat. Regimens are prolonged, associated with differing degrees of drug toxicity and success rates are variable, particularly in the treatment of drug-resistant isolates [9,10].

Personalised medicine involves tailoring the provision of healthcare to patients according to their individual characteristics and profiles in order to prevent, diagnose and treat diseases. The Council of the European Union defines personalised medicine as a medical model in which a patient’s genotypic and phenotypic data are utilised to determine their susceptibility to specified conditions, implement targeted strategies to prevent disease onset and tailor specific treatment regimens according to their needs [11]. Proponents of personalised medicine argue that it offers the benefits of enhancing prevention of disease; ensures that patients receive optimised, safer therapies that cater to their specific needs; and ultimately will improve the cost-effectiveness of healthcare services [12]. In recent years, attention has turned to the potential utility of adopting a personalised medicine approach to improve outcomes in patients with mycobacterial pulmonary infections.

**Figure 1:**
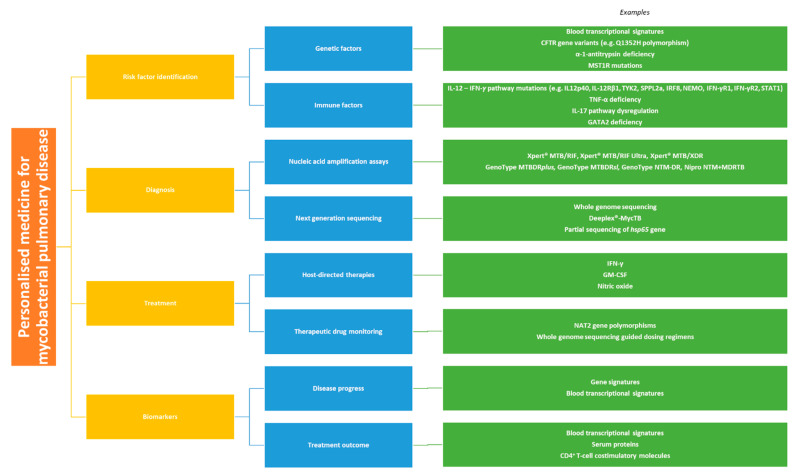
Personalised medicine approaches for the management of mycobacterial pulmonary disease.

In this review, we discuss the ways in which personalised medicine may be utilised in the management of mycobacterial lung disease. We provide an overview of how such approaches may be used to identify patients at risk of developing TB or NTM-PD, make a diagnosis of these conditions and guide effective therapeutic strategies (Figure 1). We also consider directions for future work in this field.

## 2. Identifying Risk Factors

Determining who is at risk of developing of TB and NTM-PD is important so that such patients can be monitored for the development of clinically significant disease and early preventative measures can be instituted if necessary.

### 2.1. Genetic Factors

Genome-wide association studies have identified susceptibility and protective loci for TB. In a study that combined two genome-wide association studies in populations from Ghana and The Gambia, a single nucleotide polymorphism on chromosome 18q11.2 was associated with susceptibility to TB (odds ratio (OR) 1.19, combined *P* = 6.8 × 10^−9^, 95% confidence interval (CI) 1.13–2.27) [13]. These findings however were not replicated in a meta-analysis of four studies in Chinese populations [14]. In a study in a Han Chinese population, an increased risk of TB was associated with single nucleotide polymorphisms on 14q24.3 (OR 1.277, combined *p* = 1.72 × 10^−11^) and 20p13 (OR 1.339, combined *p* = 2.37 × 10^−11^) [15]. Loci protective against TB have been identified on 11p13 [16,17]. Furthermore, in a recent study in a Vietnamese population comprising household contacts of pulmonary TB, a locus on 10q26.2 was found to be associated with protection against TB and the findings were replicated in cohorts from France and South Africa [18].

Transcriptomic studies of TB infection risk have yielded variable results. In a study of patients in Uganda, transcriptional profile analysis demonstrated that monocytes derived from patients with apparent resistance to TB infection differentially activated pathways controlled by histone deacetylases in response to *M. tuberculosis* exposure [19]. In a paediatric population in a high-incidence setting, a 51-transcript signature was shown to have a sensitivity of 82.9% and specificity of 83.6% for distinguishing between TB and other diseases [20]. In an adult population in a high-incidence setting, a 44-transcript signature was used to calculate a disease risk score for patients which was found to differentiate between active TB and other diseases with a sensitivity of 93% and specificity of 88% [21]. In a prospective study from a low-incidence setting, the highest performing transcriptomic signature indicative of active TB had both a sensitivity and specificity of 77%, which was insufficient to meet World Health Organisation (WHO) diagnostic cut-offs for non-sputum tests for smear-negative TB [22].

There is an increased risk of NTM-PD among patients with cystic fibrosis (CF). Several CF transmembrane conductance regulator (CFTR) variants have been shown to occur at a higher frequency among patients with bronchiectasis or NTM-PD than among healthy controls [23]. One prospective study found that 36% of NTM-PD patients had CFTR mutations [24]. In another study investigating familial clustering of NTM-PD, five out of 12 patients with NTM-PD secondary to various NTM species were found to have CFTR gene variants but did not have classic CF [25]. The Q1352H polymorphism of the CFTR gene has been found to occur at a higher frequency among patients with NTM-PD than among controls (OR 4.27, 95% CI 1.43–12.78) [26]. CFTR mutations have been associated with changes in DNA methylation in lung macrophages [27], increased macrophage apoptosis and decreased phagocytosis [28], all of which may contribute to immune dysregulation and thus increased NTM infection risk.

α-1-antitrypsin (AAT) deficiency may predispose to mycobacterial lung disease by decreasing phagosome-lysosome fusion in macrophages, thereby decreasing mycobacterial clearance [29]. Anomalies in AAT protein were found in 27% of patients with NTM-PD secondary to rapid-growing NTM species, which was 1.6 times higher than would be expected in the general population [30]. Pulmonary alveolar proteinosis, characterised by protein accumulation in alveoli due to defective surfactant clearance by macrophages, has also been shown to be associated with increased risk of mycobacterial infection [31,32].

Single nucleotide polymorphisms in the region of the class II human leucocyte antigen (HLA) have been shown to confer increased risk of TB infection or disease: examples include rs557011[T] and rs9271378[G], located between HLA-DQA1 and HLA-DRB1; and a missense variant (p.Ala210Thr) in HLA-DQA1 [33]. Studies on vitamin D receptor (VDR) polymorphisms and mycobacterial infection risk have yielded variable results. While one meta-analysis was inconclusive [34], a more recent analysis found that the VDR gene FokI polymorphism confers increased risk of TB, particularly among patients who are HIV-negative or Asian, but not among those who are African or Caucasian [35]. VDR polymorphisms have been associated with *M. malmoense* lung disease [36]. In a larger study however VDR TaqI and FokI polymorphisms were not found to be associated with NTM-PD susceptibility [37].

The odds of polymorphisms in the natural-resistance-associated macrophage protein 1 gene have been shown to be higher among patients with NTM-PD than non-NTM controls, although no significant differences were found in polymorphism frequency between patients with *M. avium* complex (MAC) pulmonary disease (MAC-PD) and *M. abscessus* (MAB) pulmonary disease (MAB-PD) [38]. Mutations in the macrophage-stimulating-1 receptor (MST1R) gene have been implicated in the development of “Lady Windermere syndrome”, in which NTM-PD occurs in patients who have a slender body habitus, pectus excavatum and scoliosis [39]. Activation of MST1R, a tyrosine kinase receptor on airway epithelial cells, is associated with increased ciliary function and it has been postulated that MST1R gene mutations may diminish ciliary clearance of NTM [39,40].

### 2.2. Immune Impairment

The interleukin-12 (IL-12)—interferon-γ (IFN-γ) pathway plays a pivotal role in immunity against mycobacterial disease. Mendelian susceptibility to mycobacterial disease arises due to mutations in this pathway: decreased IFN-γ production has been attributed to mutations in genes encoding IL12p40, IL-12Rβ1, TYK2 (a Janus kinase), SPPL2a (a protease), IRF8 and NEMO; whilst diminished response to IFN-γ has been attributed to mutations in genes encoding IFN-γR1, IFN-γR2, STAT1, IRF8 and NEMO [41]. A study evaluating cytokine production by peripheral blood mononuclear cells (PBMCs) found that IFN-γ, tumour necrosis factor-α (TNF-α) and IL-12 production was significantly lower in patients with NTM-PD compared to non-NTM controls [42]. A whole-blood gene expression study has shown that patients with NTM-PD exhibit reduced expression of transcripts such as *IFNG* and others involved in T-cell signalling compared to non-NTM controls [43]. Reduced *IFNG* was associated with more marked radiographic changes and altered lung function [43]. Notably polymorphisms in certain genes may be associated with one type of mycobacterial infection but not others. IL-12Rβ1 gene polymorphisms were associated with increased risk of pulmonary TB in a study in Morocco but were not associated with susceptibility to NTM-PD in a Korean population [44,45].

Deficiency in production of TNF-α, another important cytokine in antimycobacterial defence which acts via macrophage activation [46], has been associated with mycobacterial lung disease. In a study of patients with CF, the number of TNF-α-producing CD4^+^ T-cells was significantly lower among patients with current or previous MAB-PD compared to healthy controls [47]. Patients with MAC-PD have been found to exhibit diminished PBMC production of TNF-α compared to healthy controls [48]. Impaired IL-17 responses have been reported in patients with MAC-PD [49] and MAB-PD [50], while transcriptional studies have demonstrated that downstream targets of the IL-17 pathway appear to be upregulated in NTM-PD even though IL-17 itself is not [51]. Exaggerated IL-17A and Th17 responses however have been observed in active TB compared to latent TB infection (LTBI) [52].

Deficiencies in GATA2, a zinc finger transcription factor, are associated with a spectrum of haematological abnormalities such as monocytopaenia, lymphopoenias, leukaemias and susceptibility to fungal, viral and mycobacterial infections [53]. Such patients are particularly at risk of MAC infections [54].

## 3. Diagnosis

A diagnosis of TB hinges on microbiological techniques. Diagnosing NTM-PD is contingent upon consideration of patient factors, culture or PCR-based microbiology and imaging [55], as recommended by clinical guidelines [8]. Correctly identifying mycobacterial isolates and their antibiotic susceptibility is an important step in the management of mycobacterial lung disease. Phenotypic drug susceptibility testing (DST) involving culture-based techniques are widely used but their limitations include the risk of culture contamination and the time taken to yield final results. Molecular tests offer a more rapid means to establish the resistance profiles of isolates, guide the appropriate selection of antibiotics and identify transmission of isolates, with the latter being particularly important for TB outbreak control and follow up of contacts [56].

### 3.1. Nucleic Acid Amplification Assays

The Xpert^®^ MTB/RIF (Cepheid, Sunnyvale, CA, USA) is a nucleic acid amplification assay that utilises real-time polymerase chain reaction (PCR) to detect *M. tuberculosis* complex as well as mutations in the *rpoB* gene [57], which confer *M. tuberculosis* isolates with rifampicin resistance [58]. The newer Xpert^®^ MTB/RIF Ultra (Cepheid, Sunnyvale, CA, USA) improves upon the sensitivity of the Xpert^®^ MTB/RIF assay by utilising two different amplification targets and having an improved cartridge design (Table 1) [59,60]. Xpert^®^ MTB/RIF Ultra and Xpert^®^ MTB/RIF have a similar sensitivity (94.9% vs. 95.3%, respectively) and specificity (99.1% vs. 98.8%) for detecting rifampicin resistance [61]. The recently developed Xpert^®^ MTB/XDR (Cepheid, Sunnyvale, CA, USA), capable of identifying 16 resistance-conferring mutations in *M. tuberculosis* (Table 1) [62], has been shown to have a high sensitivity (94.1–100%) and specificity (100%) for detecting resistance to isoniazid, fluoroquinolones and second-line injectable drugs; the sensitivity and specificity for detecting ethionamide resistance was slightly lower (88.5% and 97.3%, respectively) [63]. Another study has shown that the assay is capable of detecting 100% of the tested mutations for resistance to these drugs but has more variable outcomes for detecting heteroresistance [64].

Line probe assays (LPAs) are DNA-DNA hybridisation assays that enable rapid detection of *M. tuberculosis* isolates and detection of multiple mutations, including in the *rpoB*, *katG* and *inhA* genes (Table 2) [65]. WHO endorses the use of first-line LPAs such as GenoType MTBDR*plus* (Hain Lifescience, Nehren, Germany) [66] and Nipro NTM+MDRTB (Nipro, Osaka, Japan) [67] to detect resistance to rifampicin and isoniazid; and the use of second-line LPAs such as GenoType MTBDR*sl* (Hain Lifescience, Nehren, Germany) to detect mutations in *gyrA*, *gyrB*, *embB, rrs* and *eis* genes that confer resistance to fluoroquinolones, ethambutol and second-line injectable agents [1]. A meta-analysis has demonstrated that GenoType MTBDR*plus*V1, GenoType MTBDR*plus*V2 and Nipro NTM+MDRTB have a high pooled sensitivity (96.7%) and specificity (98.8%) for rifampicin resistance; and slightly lower sensitivity (90.2%) but high specificity (99.2%) for isoniazid resistance [68]. In a high MDR TB incidence setting, GenoType MTBDR*sl* was found to detect resistance with a sensitivity of 73.6% for fluoroquinolones, 64.7% for ethambutol, 20% for kanamycin, 25% for amikacin and 100% for capreomycin [69]. Moreover the sensitivity and specificity of GenoType MTBDR*sl* VER 2.0 was high for fluoroquinolone resistance but more variable for resistance to second-line injectable drugs [70]. It should be noted that molecular DSTs such as Xpert^®^ MTB/RIF and GenoType MTBDR*plus* may miss certain mutations in the *rpoB* gene, such as the Ile49Phe variant seen in MDR TB in South Africa, which runs the risk of ineffective drug regimens being prescribed and further spread of infection [71].

The GenoType NTM-DR assay (Hain Lifescience, Nehren, Germany) [72] has been shown to accurately identify 100% of MAB isolates and 92.1% of MAC isolates, with certain strains of the latter being misidentified as *M. intracellulare* [73]. The same study demonstrated that GenoType NTM-DR exhibited 96.3% sensitivity and 100% specificity in detecting clarithromycin resistance; with 99.3% concordance with sequencing and 98.6% concordance with DST [73]. Additionally, the assay detected amikacin resistance with 62.5% sensitivity and 100% specificity; with 99.3% concordance with sequencing and 97.9% concordance with DST [73]. More recently, the GenoType NTM-DR assay was found to have 100% concordance with multi-locus sequence typing in identifying MAB subspecies; and 100% concordance with DST for detecting resistance to clarithromycin and amikacin [74].

### 3.2. Next Generation Sequencing (NGS)

Recently there has been an increasing shift towards investigating and establishing genotypic DST for mycobacterial disease. Advances in NGS techniques have expedited the diagnosis of mycobacterial infections. Whole genome sequencing (WGS) enables rapid identification of mycobacterial isolates and their drug susceptibility or resistance profiles, whilst also facilitating epidemiological investigations of outbreaks [75]. WGS has been shown to identify mycobacterial species with 93% accuracy, identify drug susceptibility with 93% accuracy, link isolates to outbreaks and incur 7% lower costs annually compared to routine diagnostic techniques [76]. A recent study has shown that WGS of *M. tuberculosis* DNA acquired directly from sputum was able to yield isolate resistance data up to 24 days faster than WGS of isolates cultured in a Mycobacterial Growth Indicator Tube (MGIT) and up to 31 days faster than phenotypic testing of isolates [77]. The Deeplex^®^-MycTB deep sequencing assay (Genoscreen, Lille, France) enables genotyping of *M. tuberculosis* isolates and covers 18 sequencing regions that are associated with drug resistance. It has been shown to have high concordance with phenotypic DST for first line drugs rifampicin (97.4%), isoniazid (94.9%), pyrazinamide (97.4%) and ethambutol (97.4%); and second line drugs including fluoroquinolones (66.7%), prothionamide (75.0%), aminoglycosides (100%), linezolid (100%) and bedaquiline (100%) [78].

There have been substantial advances in the genomic characterisation of previously uncharacterised NTM species in recent years due to the availability of WGS [79]. The use of WGS identified possible transmission of *M. abscessus* subsp. *massiliense* between patients with CF in a UK centre [80], but it has also been useful in refuting potential transmission of isolates between close contacts [81]. A recent study has found that there is limited concordance between WGS analysis and LPAs in the identification of mixed NTM infections [82]. Partial sequencing of the 16S rRNA gene may be insufficient to identify different NTM species [83]. Notably however partial sequencing of the *hsp65* gene from the sputum of patients with NTM-PD has been shown to identify a median 5.5 NTM species per sample compared to up to one species identified by culture-based techniques alone, implying a more diverse community of NTM species is present in the lungs than culture-based techniques would suggest [84].

## 4. Treatment

### 4.1. Host-Directed Therapies

Treatment regimens for mycobacterial infection can be tailored to address the individual clinical needs of patients according to their risk factors, comorbidities and severity of disease. One approach may be to treat specific underlying immunological deficiencies. In a case report on a patient with functional IFN-γ deficiency and NTM-PD, administration of nebulised IFN-γ1b resulted in improved NTM clearance and stabilisation of lung function [85]. In another patient with a homozygous mutation in the IFNγR1 gene and disseminated *M. avium* disease, administration of subcutaneous IFN-γ was associated with disease control and eventual regression of cerebral lesions [86]. A previous meta-analysis has demonstrated that adjunctive aerolised and intramuscular IFN-γ can improve sputum culture conversion at different time points during the course of pulmonary TB treatment [87].

In patients with disseminated NTM disease on a background of having confirmed anti-IFN-γ autoantibodies, administration of rituximab, a monoclonal antibody targeting CD20 on B cells, has been shown to reduce both the autoantibody titres and their neutralising capacity against IFN-γ; and result in clinical improvement [88,89]. The use of intravenous cyclophosphamide in patients with anti-IFN-γ autoantibodies and refractory MAB infection has had mixed outcomes [90].

Based on the hypothesis that activation of the granulocyte-macrophage colony-stimulating factor (GM-CSF) receptor enhances macrophage activation, which may be reduced in patients with CF, a case report has described how administration of inhaled GM-CSF in patients with CF and MAB-PD was associated with clinical, microbiological and radiological improvement [91]. A clinical trial found that administration of adjunctive GM-CSF in the treatment of pulmonary TB trended towards, but did not significantly enhance, sputum culture conversion at week eight of treatment [92].

Various other novel treatments have been trialled in patients with severe mycobacterial disease who have not responded to conventional therapies. A recent pilot study demonstrated that intermittent inhaled nitric oxide administration in patients with CF and refractory MAB-PD resulted in improvement in six-minute walk distance and forced expiratory volume in one second, building on the premise that higher levels of nitric oxide in CF airways are associated with improved lung function [93]. Engineered bacteriophage therapy in a patient with CF and disseminated drug-resistant *M. abscessus* infection was recently found to result in improvement in pulmonary function, liver function and dermatological lesions [94]. A range of other host-directed therapeutic strategies have been described for TB and NTM-PD, including targeting various components of the adaptive and innate immune responses [95,96,97].

### 4.2. Therapeutic Drug Monitoring (TDM)

TDM refers to the process by which medication doses are tailored to individual patients based on the drug’s blood or plasma concentration at specified times, such that the drug level is maintained within a therapeutic window that is both safe and efficacious for the patient [98]. This is important in the treatment of mycobacterial infection because inadequate dosing or drug levels that are outside the desired therapeutic window can be linked to treatment failure, drug toxicity or the emergence of drug-resistant isolates [99]. Rifampicin levels are commonly measured in clinical practice. Slow responders to TB therapy have been shown to have two-hour post-dose rifampicin levels that are below the expected range and TDM can be used to direct dose adjustment to achieve therapeutic drug levels [100].

In a study evaluating the effect of adjusting the dose of isoniazid in patients with newly diagnosed pulmonary TB according to the extent of the patients’ N-acetyltransferase 2 (NAT2) gene polymorphisms, slow acetylators receiving tailored dosing did not incur any isoniazid-induced hepatotoxicity or treatment failure; and there was a lower rate of early treatment failure among rapid acetylators receiving tailored dosing [101]. A recently developed pharmacogenomic assay has been shown to accurately detect NAT2 gene allele patterns in pulmonary TB patients and may be of use in guiding isoniazid dosing [102]. The feasibility and effect on sputum conversion rates of TDM for fluoroquinolones, which are used in the treatment of drug-resistant TB [103], is currently under investigation [104].

TDM has been used to direct dosing of drugs that have relatively narrow therapeutic windows. A retrospective evaluation in the Netherlands found that TDM-guided low doses of amikacin and kanamycin were associated with a reduced rate of ototoxicity whilst maintaining efficacy when treating MDR TB [105]. These findings were supported in a recent study in Canada in which there were low rates of ototoxicity and nephrotoxicity associated with TDM-guided low-dose amikacin in patients with MDR TB [106]. TDM has also been used to lower the dose of linezolid administered in select MDR TB patients, although the efficacy of doing so remains to be seen [107]. In settings where monitoring plasma levels of drugs may not be feasible due to resource constraints, dried blood spot (DBS) sampling may offer a practical solution. Such specimens have been shown to exhibit good concordance with conventional plasma level monitoring in TDM of linezolid when treating MDR TB [108]. DBS sampling has also been successfully used for TDM of other drugs such as rifampicin and clarithromycin [109]. Samples can be sent by post at ambient temperatures to remote reference laboratories, enabling drug doses to be tailored for patients in settings where healthcare resources may be limited [99]. Dose adjustments based on TDM combined with phenotypic or genetic DST can facilitate improved efficacy while reducing toxicity substantially in drugs that have narrow therapeutic windows.

WGS of mycobacterial isolates may hold potential in guiding dosing regimens. It has been used to quantify the degree of drug resistance that would be expected to occur for various drug resistance mutations [110]. A study on drug-resistant *M. tuberculosis* isolates in Romania identified that drug minimum inhibitory concentrations (MICs) were affected by the nature of the resistance mutation in the isolate: high MICs were associated with the *rpoB* S450L mutation for rifampicin and the *katG* S315T mutation for isoniazid [111]. DST based on WGS of drug-resistant *M. tuberculosis* isolates has demonstrated that different resistance-conferring mutations are associated with differences in MICs: for example, the MIC for isoniazid is significantly lower in isolates with the -15 c/t *inhA* promoter mutation than in isolates with the *katG* Ser315Thr mutation [112].

## 5. Biomarkers

Identifying biomarkers that are able to differentiate LTBI from TB and that can predict disease progression or therapeutic success will provide clinicians with prompt insights into how to manage patients with mycobacterial lung disease.

### 5.1. Distinguishing LTBI from Active TB Disease

Gene expression signatures indicative of LTBI are yet to be identified [113]. A recent cytokine analysis demonstrated that eotaxin, macrophage-derived chemokine and monocyte chemoattractant protein-1 were together able to differentiate between active and latent TB with a sensitivity of 87.8% and specificity 91.8% [114]. In patients who are IFN-γ release assay (IGRA) positive but acid-fast bacilli negative, signatures of HLA-DR^+^IFN-γ^+^ CD4^+^ T-cells and CD45RA^-^CCR7^-^CD127^-^IFN-γ^-^IL-2^-^TNF-α^+^ CD4^+^ T-cells were able to distinguish between active TB and LTBI [115]. A whole blood gene signature comprising the genes *GBP5*, *DUSP3* and *KLF2* has been shown to distinguish between LTBI and active TB in a multicohort analysis [116]. Furthermore various transcriptomic signatures that distinguish between latent and active disease in high-incidence settings have been reported [20,21]. Recently however in a whole blood microarray analysis of TB patients in a low-incidence setting, transcriptomic signatures were not found to be sufficiently sensitive or specific to diagnose TB [22]. Additionally, a review has found that the diagnostic accuracy of previously published transcriptomic signatures for TB was lower than reported [117].

### 5.2. Predictors of Disease Progression

Prospective studies have identified unique gene and transcriptomic signatures predictive of TB progression. In a study of adolescents infected with *M. tuberculosis*, a 16 gene signature was shown to identify risk of TB progression with a sensitivity of 66.1% and specificity of 80.6% in the year preceding TB diagnosis [118]. More recently whole blood transcriptomic and proteomic analyses have demonstrated increased type I/II IFN signalling 18 months preceding TB diagnosis and suppression of Th17 responses in patients progressing from infection to active pulmonary disease [119].

### 5.3. Predictors of Treatment Outcome

A blood transcriptional signature for active TB that correlates with radiological changes has previously been described and shown to change to that of healthy controls following TB treatment [120]. Blood transcriptional signatures for active TB and treatment response have been shown to attenuate over the course of treatment, particularly following the initial two weeks of treatment [121]. Various other biomarkers of TB treatment response have been identified, including serum proteins such as C-reactive protein, IL-1β, IL-6, matrix metalloproteinase-8 (MMP-8), procalcitonin, pentraxin 3 and serum amyloid A1, all of which were strongly associated with baseline TB severity and modulated by TB treatment [122]. A range of costimulatory molecules in CD4+ T-cells have been implicated in active TB and suggested as being useful for monitoring response to treatment. Active TB has been associated with a CD38^positive^CD27^low^ CD4^+^ T-cell phenotype, treated TB with a CD38^negative^CD27^low^ phenotype and latent TB with a CD38^negative^CD27^high^ phenotype [123]. Persistent culture positivity has been associated with serum RANTES level at the time of diagnosis and MMP-8 levels following two months of treatment [124]. Urinary lipoarabinomannan (LAM), which has been shown to reduce 8-week mortality when it is used to guide initiation of anti-TB therapy in patients with HIV infection and suspected TB [125], may also have a role in monitoring treatment response. Among patients with culture-confirmed pulmonary TB in a high-incidence setting, a strongly positive urinary LAM following two months of intensive therapy was found to be associated with a significantly higher risk of mortality during three-year follow-up than a weakly positive or negative urinary LAM result [126].

Microbiological biomarkers of treatment response in macrolide-susceptible MAC-PD may include the time to positivity (TTP) for MGIT culture systems, with one recent retrospective study identifying that a TTP of greater than seven days at baseline and a TTP of greater than 15 days following three months of treatment were predictive of sputum culture conversion within the initial six months of therapy [127]. Potential blood biomarkers of treatment response in MAC-PD include a decrease in serum anti-glycopeptidolipid IgA levels following treatment; a worsening in radiographic changes has been observed in those patients in whom antibody levels rose following treatment cessation [128]. Th1-related cytokine levels have been shown to be reduced at the time of MAC-PD diagnosis but improve following 12 months of treatment, while Th-17 related cytokines have been associated with failure of sputum conversion [129]. Moreover four differentially expressed serum miRNAs implicated in host immune response have recently been identified as being expressed at higher levels among NTM-PD patients than healthy controls, earmarking these as additional potential biomarkers for diagnosis and treatment response [130].

Biomarkers capable of reliably guiding an individualised duration of anti-mycobacterial therapy are needed, particularly in the treatment of drug-resistant isolates. This is important as a ‘one size fits all’ approach to treatment length runs the risk of many patients being exposed to the toxicity of therapy for longer than may be clinically needed. Notably a whole blood transcriptomic model evaluated in cohorts in Germany and Romania has recently been shown to predict the duration of treatment required in patients with TB; and found that cure could be achieved in most patients with MDR TB using a shorter treatment duration [131]. Any approaches to individualise duration of therapy will require validation in large cohorts of patients.

## 6. Future Priorities

In recent years, there have been significant advances in the development personalised medicine approaches to identify those at risk of mycobacterial lung disease, obtain a definitive diagnosis and tailor therapies to meet individual patients’ needs. There remain however a number of challenges if such approaches are to have a meaningful impact on the clinical management of TB and NTM-PD globally. Widespread implementation of personalised medicine will be contingent upon having the prerequisite infrastructure in place, which may be particularly difficult in resource-limited settings; although as illustrated by the use of DBS in TDM, novel solutions may be found. Investment will be needed both in health technologies and training of staff for effective scale up to be achieved, requiring political buy-in and visionary leadership from health policymakers.

Further clinical studies and trials are needed to establish reliable biomarkers that not only indicate increased host susceptibility to mycobacteria, but also are able to distinguish between innocuous colonisation, infection and active disease; discriminate between mild and more severe disease courses; predict treatment response and duration; and predict the likelihood of future relapse or reinfection. There remains an imperative to develop diagnostic systems with fast turn-around times that are based on rapid molecular technologies in order to facilitate tailoring of therapy for mycobacterial lung disease [132]. While IGRAs enable identification of latent TB infection, they do not provide information on which patients are at risk of progression to active disease and interpreting the significance of borderline or indeterminate results can be complex [133]. The role of digital technologies in supporting treatment adherence in mycobacterial disease has been studied and combining data from such digital tools with personalised medicine data may yield new insights into likely disease outcomes. It will also be important to monitor therapeutic responses using validated patient-reported outcome measures [134]. Integrating artificial intelligence with personalised medicine techniques will have a transformational effect on clinicians’ abilities to combine and process large volumes of genomic, clinical, microbiological and radiological data in order to individualise management plans [135]. This in turn will facilitate greater targeted monitoring of patients who are at highest risk of clinical deterioration through enhanced follow up or more intensive therapy. Ultimately a clear framework for the application of personalised medicine in mycobacterial pulmonary disease will be needed to facilitate its uptake in routine clinical practice.

## Figures and Tables

**Table 1:** Nucleic acid amplification tests for detecting resistance in *M. tuberculosis* isolates [59,60,62].

Nucleic Acid Amplification Test	Antibiotic Resistance Detected	Corresponding Mutations
Xpert^®^ MTB/RIF	Rifampicin	*rpoB*
Xpert^®^ MTB/RIF Ultra		
Xpert^®^ MTB/XDR	Isoniazid	*katG*, *fabG1*, *oxyR-ahpC* intergenic region, *inhA* promoter
	Ethionamide	*inhA* promoter
	Fluoroquinolones	*gyrA* and *gyrB* quinolone resistance determining regions
	Second-line injectable drugs	*rrs*, *eis* promoter

**Table 2:** Line probe assays for detecting resistance in mycobacterial isolates [66,67,72].

Line Probe Assay	Mycobacterial Isolates Detected	Antibiotic Resistance Detected	Corresponding Mutations
GenoType MTBDR*plus* VER 2.0	*M. tuberculosis*	Rifampicin	*rpoB*
		Isoniazid	*katG*, *inhA* promoter
GenoType MTBDR*sl* VER 1.0		Ethambutol	*embB*
		Fluoroquinolones	*gyrA*
		Second-line injectable drugs	*rrs*
GenoType MTBDR*sl* VER 2.0		Fluoroquinolones	*gyrA*, *gyrB*
		Second-line injectable drugs	*rrs*, *eis*
Nipro NTM+MDRTB detection kit 2	*M. tuberculosis* complex and differentiates *M. avium*, *M. intracellulare & M. kansasii*	Rifampicin	*rpoB*
		Isoniazid	*katG*, *inhA*
GenoType NTM-DR	NTM	Macrolides	*rrl*, *erm*(41) (in MAB only)
		Aminoglycosides	*rrs*
